# Improved 3D tumour definition and quantification of uptake in simulated lung tumours using deep learning

**DOI:** 10.1088/1361-6560/ac65d6

**Published:** 2022-04-27

**Authors:** Laura Dal Toso, Zacharias Chalampalakis, Irène Buvat, Claude Comtat, Gary Cook, Vicky Goh, Julia A Schnabel, Paul K Marsden

**Affiliations:** 1 School of Biomedical Engineering and Imaging Sciences, King’s College London, London, United Kingdom; 2 Laboratoire d’Imagerie Biomédicale Multimodale (BioMaps), Université Paris-Saclay, CEA, CNRS, Inserm, Service Hospitalier Frédéric Joliot, Orsay, France; 3 ABX-CRO Advanced Pharmaceutical Services Forschungsgesellschaft M.B.H., Dresden, Germany; 4 Laboratoire d’Imagerie Translationnelle en Oncologie, Inserm, Institut Curie, Orsay, France; 5 Helmholtz Center Munich, Munich, Germany; 6 Faculty of Informatics, Technical University of Munich, Munich, Germany

**Keywords:** PET, CNN, quantification

## Abstract

*Objective.* In clinical positron emission tomography (PET) imaging, quantification of radiotracer uptake in tumours is often performed using semi-quantitative measurements such as the standardised uptake value (SUV). For small objects, the accuracy of SUV estimates is limited by the noise properties of PET images and the partial volume effect. There is need for methods that provide more accurate and reproducible quantification of radiotracer uptake. *Approach.* In this work, we present a deep learning approach with the aim of improving quantification of lung tumour radiotracer uptake and tumour shape definition. A set of simulated tumours, assigned with ‘ground truth’ radiotracer distributions, are used to generate realistic PET raw data which are then reconstructed into PET images. In this work, the ground truth images are generated by placing simulated tumours characterised by different sizes and activity distributions in the left lung of an anthropomorphic phantom. These images are then used as input to an analytical simulator to simulate realistic raw PET data. The PET images reconstructed from the simulated raw data and the corresponding ground truth images are used to train a 3D convolutional neural network. *Results.* When tested on an unseen set of reconstructed PET phantom images, the network yields improved estimates of the corresponding ground truth. The same network is then applied to reconstructed PET data generated with different point spread functions. Overall the network is able to recover better defined tumour shapes and improved estimates of tumour maximum and median activities. *Significance.* Our results suggest that the proposed approach, trained on data simulated with one scanner geometry, has the potential to restore PET data acquired with different scanners.

## Introduction

Positron emission tomography (PET) is an imaging modality which is extensively used in oncology to detect and stage tumours, and to monitor response to treatment. In clinical routine, image interpretation is usually performed by visual inspection of PET images, which leads to inter- and intra-observer variability. Although in many cases visual assessment may be sufficient, more challenging tasks such as the evaluation of the response to therapy of solid tumours require some form of quantification (Boellaard *et al* 2004). In PET, uptake quantification is usually performed using semi-quantitative measurements of tumour radiotracer uptake, such as the standardised uptake value (SUV). The SUV is defined as the ratio of the radioactivity concentration in a region of interest (ROI) to the average radioactivity concentration in the whole body and can be calculated as follows:

\begin{eqnarray*}\mathrm{SUV}=\frac{\mathrm{Activity}\,{\mathrm{Concentration}}_{\mathrm{ROI}}\,(\mathrm{kBq}\,{\mathrm{ml}}^{-1})}{\mathrm{Injected}\,\mathrm{Activity}\,(\mathrm{MBq})\,/\,\mathrm{Body}\,\mathrm{Weight}\,(\mathrm{kg})}.\end{eqnarray*}

Usually a normalisation by a mass density of 1 (g ml^−1^) is assumed, and SUV is presented as a dimensionless metric. Different SUV metrics, such as SUV_max_, SUV_mean_ and SUV_peak_ can be defined. These semi-quantitative metrics are affected by the noise properties of PET images as well as partial volume effects (PVE), which compromise the accuracy and reproducibility of the measurements (Boellaard *et al* 2004, Zaidi and Karakatsanis 2017). The PVE, which results from the poor spatial resolution of PET scanners and from image sampling, degrades the quantitative accuracy of PET images and it can result in large biases on measures of tracer uptake in small tumours (Soret *et al* 2007, Cysouw *et al* 2017). Most commonly SUV_max_, which is calculated using the most intense tumour voxel within the ROI, is reported. SUV_max_ is widely used as it is user-independent, but it is also affected by noise. A metric that is less dependent on noise is SUV_mean_, but its disadvantage is that it depends on the delineation of the volume of interest (VOI) in which the measurement is performed. SUV_peak_ measures the average activity concentration within a VOI. Various definitions of SUV_peak_ using different VOI shapes, sizes and locations can be found in literature (Vanderhoek *et al* 2012). In this paper, the maximum SUV_peak_ value measured in a 1 cm^3^ spherical VOI within the tumour is reported. SUV_peak_ is less affected by image noise than SUV_max_, but it presents some issues when applied to small tumours, especially if they are smaller than the VOI in which the peak is measured.

In this paper, PET images are reconstructed using the ordered subsets expectation maximisation (OSEM) algorithm. To improve PET image quality a model of the system resolution can be integrated in the reconstruction algorithm. Resolution modelling typically results in enhanced images where small lesions and narrow structures are characterised by a better contrast. Although resolution modelling can significantly improve image resolution and contrast it can also introduce artefacts near sharp edges in the PET image. These artefacts resemble the familiar Gibbs artefact and can lead to overestimation of quantitative indices—this is a particular issue for small lesions where PSF-reconstruction can lead to enhanced detectability but poor quantitative accuracy including overestimation of SUV max and peak indices.

There is need for reproducible methods that allow for more accurate quantification of tumour radiotracer uptake. Improved uptake quantification would lead to a more accurate assessment of response to treatment, especially at the early stages.

The use of artificial intelligence in the field of medical imaging has increased dramatically over the last decade. In PET imaging, machine learning and deep learning methods have been successfully applied to tumour segmentation, classification, automatic detection and image reconstruction (Gong *et al* 2020, Kim *et al* 2018, Litjens *et al* 2017, Shiyam Sundar *et al* 2021). In recent years, deep learning methods have been used to denoise static PET images, and they have demonstrated better performance than traditional denoising approaches for various tracers and tasks. The two main deep learning architectures that have been used for denoising are convolutional neural networks (CNNs) (Gong *et al* 2019) and generative adversarial networks (Wang *et al* 2018). In previous work we developed a deep learning algorithm using a 3D CNN with the aim to improve quantification of tumour radiotracer uptake in simulated PET images (Dal Toso *et al* 2019). The network was trained on simulated ‘ground truth’ images that presented 3D shapes with typical tumour activity distributions found in clinical FDG images and on a corresponding set of simulated PET images. The network was able to robustly estimate the original activity, yielding improved images in terms of shape, activity distribution and quantification of activity. The main limitation of our previous work was that the PET images were simulated in a simplistic way, which did not take into account many of the effects that degrade the image quality in PET images.

Supervised deep learning methods require large amounts of labelled data, which are hard to obtain in PET imaging. Usually PET studies only comprise a relatively small number of patients. Furthermore, the true radionuclide distribution, which would correspond to the ‘label’ of the PET images, is very difficult to obtain and rarely known. This limitation affects not only deep learning-based methods, but all the PET data processing methods (i.e. image reconstruction) which can never be fully evaluated *in vivo*. The use of phantoms and realistic PET simulators partially overcomes the lack of large labelled datasets. Monte Carlo simulation is the most commonly used technique to generate realistic PET data, but it has the disadvantage of being computationally very demanding. A number of analytical simulators have been developed to generate simulated PET images in a shorter time (Berthon *et al* 2015, Pfaehler *et al* 2018). While analytical simulators are not as accurate as Monte Carlo based simulators, they enable fast generation of PET data with realistic noise properties, which makes them particularly useful for creating large numbers of datasets. The simulation of realistic tumours also has some limitations. Real tumours are often characterised by inter- and intra- tumour heterogeneity, and by complex spatial structures. The mathematical and computational models of cancer that have been implemented so far mainly focus on describing a few specific aspects of the disease (Bekisz and Geris 2020). These models are not able to capture all the characteristics of tumour biology. In clinical practice, biopsies are performed to obtain ground truth information on tumour composition, but this method only provides limited information as the samples are extracted from a small region, and they cannot provide a description of the whole tumour. In this work, we generated a dataset of tumours assigned with three different activity patterns. The simulated dataset offers a wide variety of tumour activity values and tumour shapes, located at different positions within the left lung of an anthropomorphic phantom. This dataset is then used to train and test a 3D CNN that can recover the real activity distribution from the signal seen on the PET image. We build on our previous deep learning approach described in Dal Toso *et al* (2019) by significantly enhancing the simulation of PET images using an analytical simulator developed by Stute *et al* (2015) and by testing the proposed approach on three datasets simulated with different characteristics.

## Material and methods

**Figure 1.**
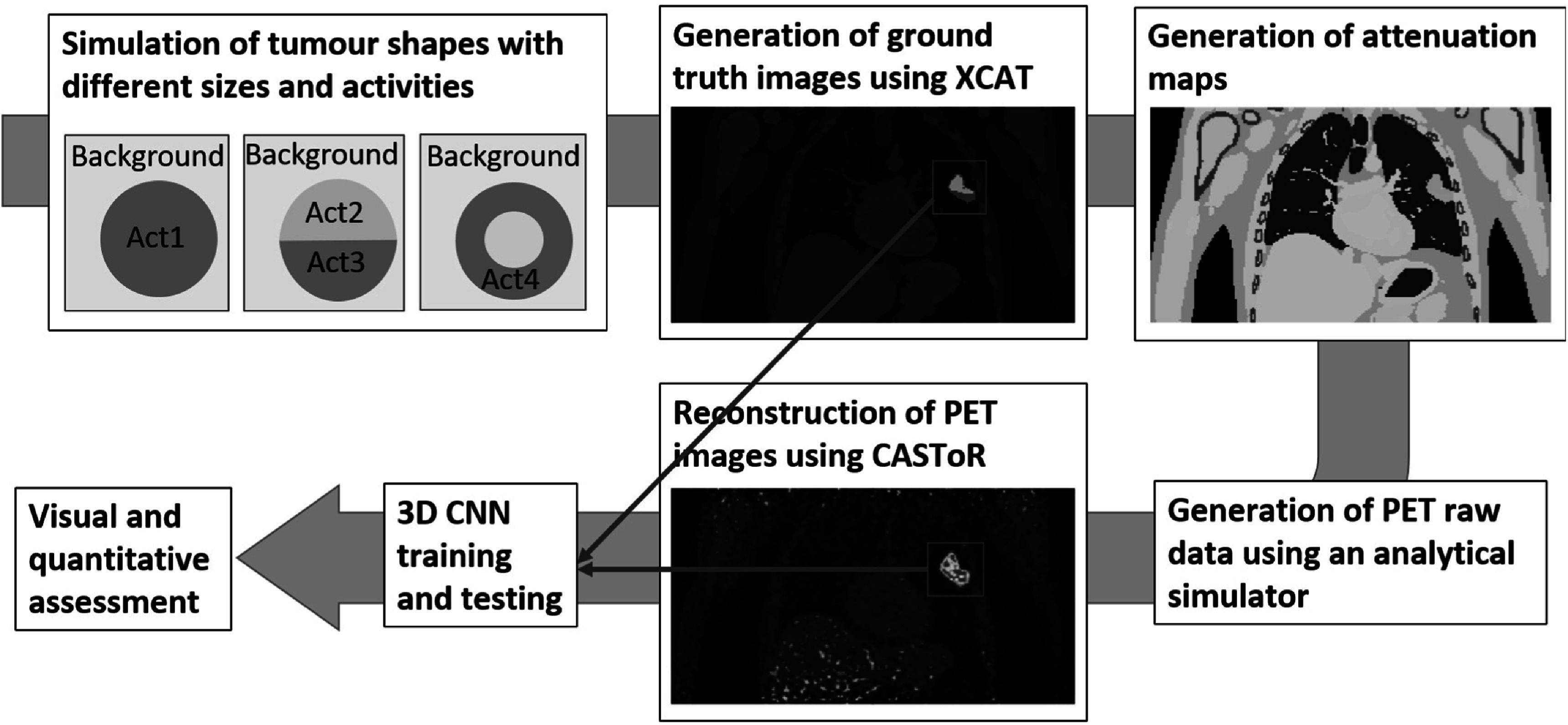
This figure shows an overview of the proposed method. Simulated tumours with various sizes and activities are simulated, and placed in random positions in the left lung of an anthropomorphic phantom, to generate the ground truth images. The attenuation maps are created, and used as input to an analytical simulator to generate the PET raw data. PET images are then reconstructed using CASToR (Merlin *et al* 2018). 3D regions are cropped around the tumours and used to train a 3D CNN, which is then tested on an unseen set of reconstructed PET images.

An overview of the proposed deep learning approach in presented in figure 1. Tumours with different sizes, shapes and activity distributions were simulated and subsequently placed in the left lung of an anthropomorphic phantom (Segars *et al* 2010). Different activities were assigned to each organ to create the ‘ground truth’ images. Additionally, the corresponding attenuation maps were created. These two were used as input to an analytical PET simulator to generate PET raw data, which were subsequently reconstructed to provide the simulated PET images. The ground truth images and simulated PET images were used to train a 3D CNN.

### Simulation of ground truth images

At first, tumour-like 3D shapes with different volumes, spanning 0.01–200 ml, were simulated. This set of tumours was composed of three groups, each corresponding to a different activity pattern: tumours filled with uniform activity, tumours split into halves (each assigned with a different activity), and hollow tumours with background activity in the inner part, to mimic necrotic regions. The ratio between the activities assigned to the two halves of the heterogeneous tumours was variable, and the tumour activities ranged from 6 to 35 kBq ml^−1^. The same activity range was used for the uniform tumours and for the hollow tumours. The thickness of the external layer of hollow tumours was set to half the radius of the tumour, and the inner core of the tumour was assigned with background activity. The background activity was set to 1/10th of the maximum tumour activity, so in each image the tumour to background ratio was equal to 10. The choice of this specific set of activity patterns was based on the work of Pfaehler *et al* (2018), in which realistic phantom inserts were designed according to non-small cell lung cancer tumours extracted from patient studies. The simulated tumours were subsequently placed in random positions into the left lung region of the XCAT phantom (Segars *et al* 2010). In order to shorten the simulation times, the XCAT phantom was reduced to 344 × 344 × 127 voxels including only the torso. The voxel size of the cropped image was [2.09, 2.09, 2.03] mm^3^. Realistic activity values drawn from a range of radioactivity concentrations measured from real patients PET images were assigned to each simulated tumour. Attenuation maps were generated for each XCAT image using the XCAT phantom attenuation values.

### PET data simulation and reconstruction

To generate realistic PET raw data we used an analytical PET simulator developed by Stute *et al* (2015). The geometry, detector resolution, and sensitivity of a positron emission tomography/magnetic resonance (PET/MR) system, more specifically the Siemens mMR scanner (Siemens Biograph mMR, Erlangen, Germany), were used in the simulation. In this work only the PET component of the simulator was modelled and we did not use any anatomical information from the MR. PET raw data were generated from the ground truth images each with 100 million total prompt counts, including scatter and random events. This corresponds to 12.4 million noise equivalent counts. Subsequently, image reconstruction was performed using the open source fully quantitative reconstruction platform CASToR (Merlin *et al* 2018), with an iterative OSEM algorithm run to 6 iterations with 21 subsets. The reconstructed voxel size was [2.09, 2.09, 2.03] mm^3^, as in the XCAT phantom images. Image reconstruction was performed with image-based PSF modelling, which is known to lead to increased SUV measurements (i.e. ${{\mathrm{SUV}}}_{{\mathrm{\max }}}$ and SUV_mean_) especially for small lesions (Lasnon *et al* 2012). No filter was applied to the images post reconstruction. Two different datasets were generated using the analytical simulator. The first one, called dataset 1, was composed of 800 uniform tumours, 721 tumours split in half and 589 hollow tumours. In this case the analytical simulator generated the PET data with an anisotropic, spatially invariant PSF with FWHM (4.5, 4.5, 4.0) mm, which was also used in the reconstruction of the simulated PET images. A total of 2110 simulated PET raw datasets and images were generated.

One of the aims of this work was to test if the proposed algorithm trained on a given dataset could generalise well to data acquired with different scanners. One way to simulate the diversity between different scanners is to generate and reconstruct PET images using a range of PSFs. As a result, a new dataset (called dataset 2) composed of 100 images, with 33 uniform tumours, 34 tumours in halves and 33 hollow tumours was generated using a range of anisotropic spatially invariant PSFs. A value randomly drawn from a Gaussian distribution (mean *μ* = 4.5 mm and sigma *σ* = 0.2 mm) was assigned to the transaxial components of the PSF, both in the simulation and in the reconstruction, with a perfect match. The axial component was calculated by dividing this value by 1.125 and a constant ratio between transaxial and axial PSF components was maintained.

### Network architecture

**Figure 2.**
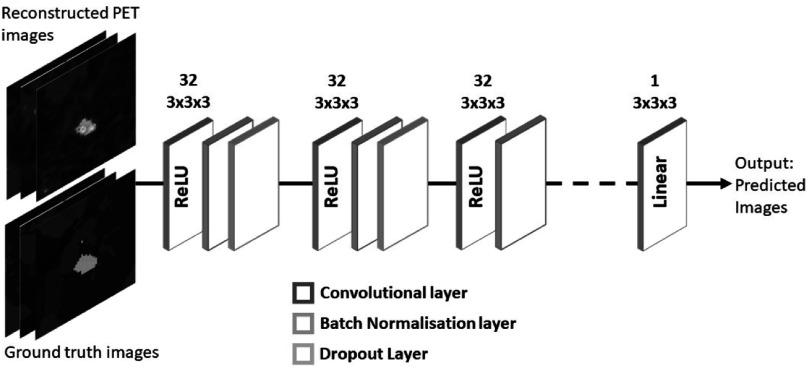
Reconstructed PET images and ground truth images, made of 50 × 50 × 50 voxels, were used as input to the 3D CNN for training. Once tested on an unseen set of reconstructed PET images the network yielded the corresponding predicted images.

CNNs are among the most commonly used algorithms for medical imaging applications (Yamashita *et al* 2018). These networks are composed of a series of convolutional layers, which can extract features from the input images, at multiple levels of abstraction. In this work 3D CNNs with different depths were tested and a visual and quantitative assessment suggested that a 3D CNN with 7 convolutional layers yielded the best results on our dataset. The proposed 3D CNN, presented in figure 2, is composed of 7 convolutional layers each followed by a batch normalisation layer except for the final layer. The convolutional layers are characterised by 32 filters with dimensions 3 × 3 × 3, and by ReLU activation functions except for the final one which has linear activation function. Two dropout layers, with dropout rate 0.3, were added after the first and second batch normalisation layers. Mean squared error was used as loss function during training and the optimizer was Adam (Kingma and Ba 2014). The learning rate was set to the default value 0.001. The training was performed using a NVIDIA Tesla K40 GPU and the network architecture was implemented in the Keras Framework with Tensorflow (Abadi *et al* 2016).

### Experiments

All the simulated PET images were cropped around the tumours to a final dimension of 50 × 50 × 50 voxels, before being processed by the CNN. The network inputs were normalised using the MinMaxScaler, which is a function provided in the scikit-learn Python package (Pedregosa *et al* 2011). Using this function, the training, testing and validation datasets were scaled in the range [0,1]. The normalisation factors were stored and subsequently applied to the network’s predictions to rescale the resulting images, before performing any quantitative analysis. In all experiments, a visual assessment of the predicted images was at first performed using a free software tool for multimodality medical image analysis (AMIDE) (Loening and Sam Gambhir 2003). Subsequently, the images were quantitatively assessed. In order to provide a baseline comparison we used multiple metrics for the quantitative assessment of the results. The maximum, median and peak values were estimated for each tumour in order to quantitatively compare the reconstructed PET images to the images predicted by the CNN. Tumour masks defined on the ground truth images were subsequently applied to the reconstructed PET images and to the CNN’s predicted images to measure the median and peak values. To present the results in a more compact way, the recovery coefficients (RCs) defined as the ratio between the observed activity and the ground truth activity, were calculated using the maximum, median and peak values as shown in equations (2), (3) and (4) respectively

\begin{eqnarray*}{{\mathrm{RC}}}_{\max }=\displaystyle \frac{{\mathrm{Max}}\,{{\mathrm{activity}}}_{{\mathrm{prediction}}}}{{\mathrm{Max}}\,{{\mathrm{activity}}}_{{\mathrm{ground}}\,{\mathrm{truth}}}},\end{eqnarray*}

\begin{eqnarray*}{{\mathrm{RC}}}_{{\mathrm{median}}}=\displaystyle \frac{{\mathrm{Median}}\,{{\mathrm{activity}}}_{{\mathrm{prediction}}}}{{\mathrm{Median}}\,{{\mathrm{activity}}}_{{\mathrm{ground}}\,{\mathrm{truth}}}},\end{eqnarray*}

\begin{eqnarray*}{{\mathrm{RC}}}_{{\mathrm{peak}}}=\displaystyle \frac{{\mathrm{Peak}}\,{{\mathrm{activity}}}_{{\mathrm{prediction}}}}{{\mathrm{Peak}}\,{{\mathrm{activity}}}_{{\mathrm{ground}}\,{\mathrm{truth}}}}.\end{eqnarray*}

When testing the network on simulated data, the structural similarity index measure (SSIM) was also calculated. The structural similarity allows the comparison of two images by taking into account their luminance *l*, contrast *c* and structure *s*. The SSIM is defined as:

\begin{eqnarray*}{\mathrm{SSIM}}(x,y)={[l(x,y)]}^{\alpha }\,\cdot \,{[c(x,y)]}^{\beta }\,\cdot \,{[s(x,y)]}^{\gamma },\end{eqnarray*}

where *x* and *y* are two non-negative image signals, which could be for example two image patches. *α*, *β* and *γ* are used to adjust the relative importance of the three components. In this paper, *α*, *β* and *γ* are equal to 1. The luminance comparison function *l* between two image signals depends on the mean intensities of the two signals *μ*_*x*_ and *μ*_*y*_. After estimating the luminance, the mean intensity is removed from the initial image signal. At this point, contrast comparison between the two signals is performed by measuring their standard deviations *σ*_*x*_ and *σ*_*y*_. Finally, each signal is normalised by its standard deviation and the structure comparison is made on the normalised signals. In practice, when two images (*X*,*Y*) are compared, the overall image quality can be estimated using the MSSIM, which it is expressed as:

\begin{eqnarray*}{\mathrm{MSSIM}}(X,Y)=\displaystyle \frac{1}{M}\sum _{j=1}^{M}({\mathrm{SSIM}}({x}_{j},{y}_{j})),\end{eqnarray*}

where *x*_*j*_ and *y*_*j*_ are the image contents at the *j*th local window and *M* is the number of local windows in the image. In this work, the MSSIM was used to compare the shape and texture of the reconstructed PET images and the predicted images to the ground truth images. The window used to estimate the MSSIM were composed of 7 × 7 × 7 voxels.

#### CNN training and testing using PET data generated with a single PSF

This experiment was performed to optimise the network’s architecture and test its performance on the simulated data. Dataset 1 was used to train and test the network. In this dataset, the PET images were generated and reconstructed using a spatially invariant PSF with FWHM (4.5 mm, 4.5 mm, 4.0 mm). The simulated images were split into training and testing datasets, with ratio 80/20. 20 % of the training data were used for validation. The training dataset was augmented by scaling some of the bigger tumours with scaling factors ranging from 0.5 to 0.8, with the aim to increase the number of small tumours. As a result, the training dataset was composed of 645 uniform tumours, 645 tumours split in halves (of which 70 were augmented) and 645 hollow tumours (of which 177 were augmented). In this experiment, the network was trained for 500 epochs with batch size 50. The test dataset was made of 422 non-augmented images. This experiment is henceforth referred to as experiment 1.

#### Application to PET data generated with different PSFs

The second aim of this work was to test if the proposed 3D CNN could generalise well to PET data generated with different PSFs and restore these images accurately. In this experiment the 3D CNN was trained on dataset 1 generated with one PSF, and subsequently applied to dataset 2, generated with a range of PSFs which were not learned during training. This experiment is henceforth referred to as experiment 2.

#### Application to PET data generated with different noise levels

As a proof of concept, the proposed 3D CNN was applied to two small datasets characterised by two different noise levels. These two datasets were generated starting from the same ten ground truth hollow tumours. These tumours were assigned with activity concentrations ranging from 6 to 35 kBq ml^−1^, and they all had volumes larger than 5 ml. Two sets of PET images were generated from the same ground truth images, using the analytical simulator. This was done by setting the number of total prompts to 50 millions and to 200 millions respectively. Both datasets were generated using a spatially invariant PSF with FWHM (4.5, 4.5, 4.0) mm. The CNN, trained on PET images simulated with 100 million total prompt counts, was applied to the two small datasets generated with different statistics. This experiment is henceforth referred to as experiment 3.

## Results

In this section, the results obtained training and testing the 3D CNN on data generated with a single PSF are presented. Subsequently, the same network is applied to PET data generated with a range of PSFs and the results are qualitatively and quantitatively compared to those obtained in the first experiment.

### CNN training and testing using PET data generated with a single PSF

**Figure 3.**
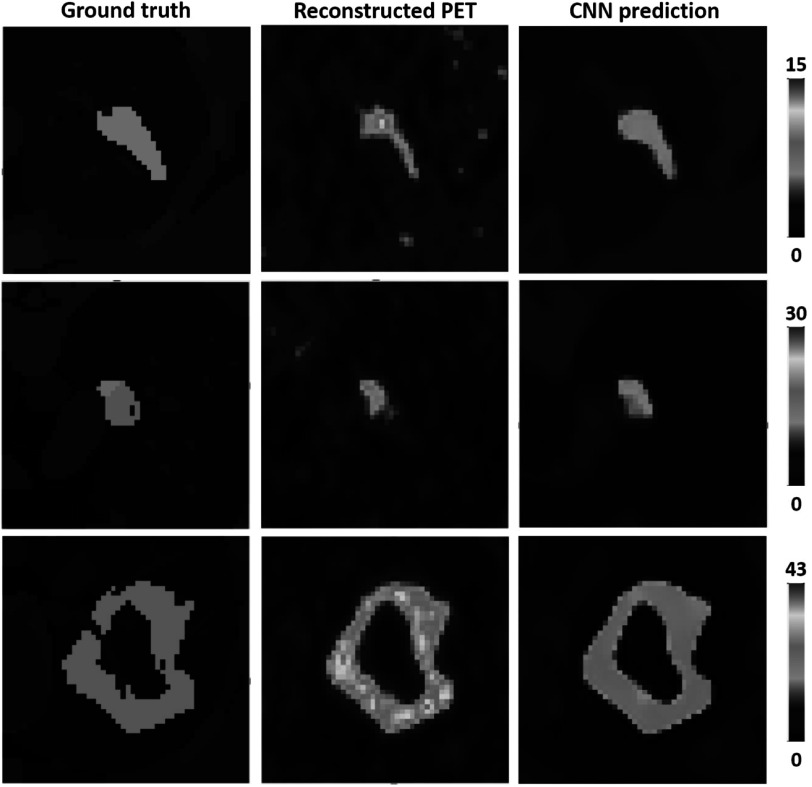
Transverse views of three representative volumes, belonging to experiment 1, where the reconstructed PET images were generated with one PSF. Each column shows the ground truth images, the reconstructed PET images and the CNN’s predicted images respectively. In each row the images are shown with the same colour scale expressed in kBq ml^−1^.

**Figure 4.**
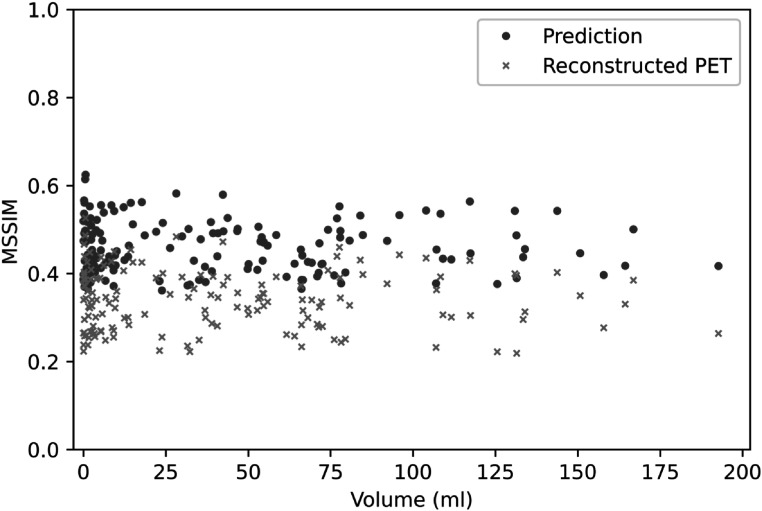
This figure shows 150 representative MSSIM values, obtained in experiment 1, where the reconstructed PET images were generated with one PSF. The MSSIMs between reconstructed PET images and ground truth images are shown in orange, the MSSIMs between predictions and ground truth are plotted in blue.

**Figure 5.**
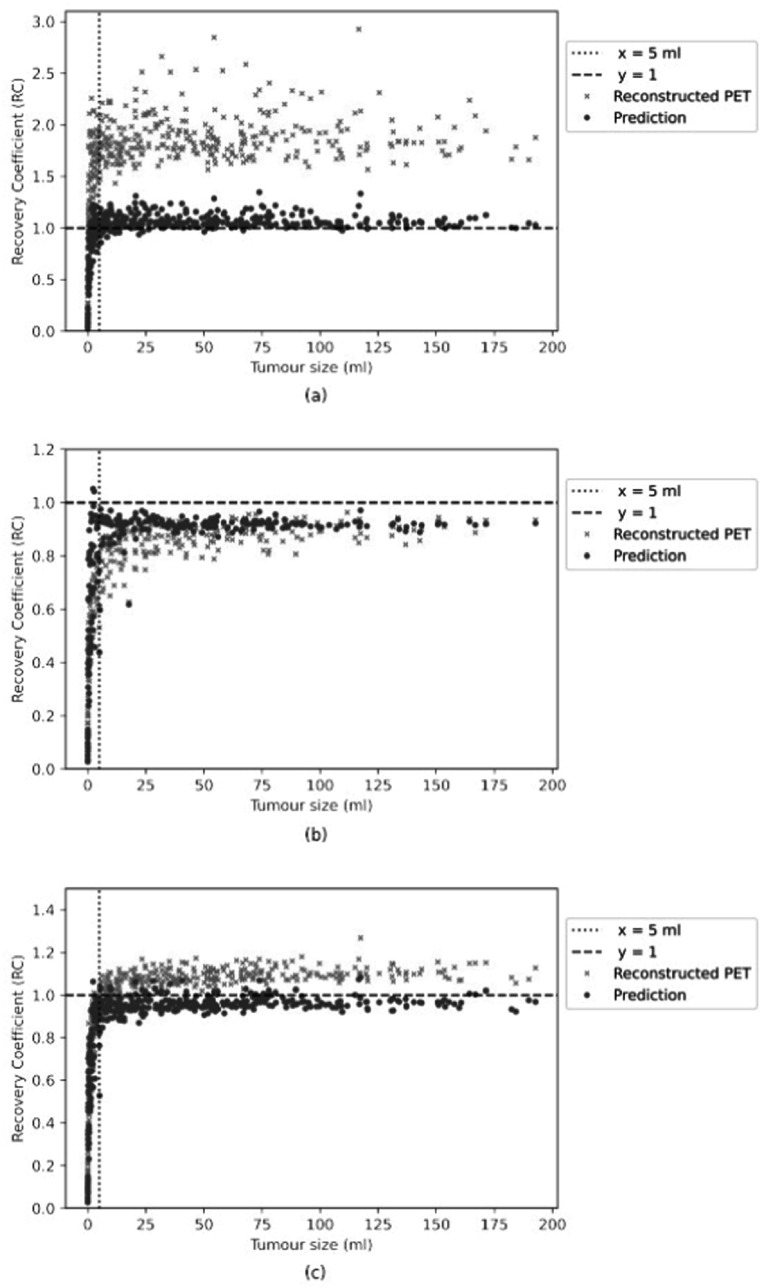
The RC_max_ and RC_median_ values, obtained training and testing the network on PET data generated with a single PSF, are plotted against the tumour volume in (a) and (b) respectively. The RC_peak_ values measured on the same dataset are shown in (c). The coefficients measured using the reconstructed PET images are shown in orange, whereas the ones measured using the predicted images are shown in blue. Tumours split in halves, each assigned with a different activity, were excluded from the calculation of the median values.

The first experiment was performed using the simulated training and test datasets generated with a single PSF. Three representative volumes, each characterised by a different activity pattern, are presented in figure 3. The CNN yielded better defined tumour shapes and denoised images in all three cases. This visual assessment was followed by a quantitative analysis. In figure 4, 150 randomly selected MSSIM values are shown. The average MSSIM value measured using the ground truth images and the reconstructed PET images is 0.33 ± 0.06. An improved average MSSIM value equal to 0.47 ± 0.06 is measured using the CNN predictions and the ground truth images. The predicted images are overall characterised by higher MSSIM values. To further assess the performance of the network, we then measured the maximum, median and peak RCs. The median value was only estimated for the tumours with uniform uptake and for the hollow tumours, which also had uniform uptake. In figure 5 the RCs are plotted against the tumour volume expressed in ml. The maximum, median and peak RC were not well recovered for tumours that have a volume smaller than around 5 ml, so we performed a detailed investigation of the small tumours. Out of all the test images, the network did not recover any increased uptake for 5 tumours, which had a volume smaller than 0.18 ml (20 voxels). In these predicted images no tumour could be detected. The tumour activities were not accurately recovered for tumours with volumes between 0.18 and 1.33 ml that were located close to other structures (ribs, heart) and that were characterised by a low ground truth activity. To better describe the RC curves we present two separate measurements, one describing the recovery of bigger volumes and the second measured for smaller volumes. The first metric we used is the average RC value, measured only for tumours with volume exceeding 5 ml. These average RC_max_, RC_median_ and RC_peak_ values measured on the reconstructed PET images and on the CNN predictions are summarised in table 1. All the RCs measured for the predicted images are approaching 1, meaning that the CNN yields improved estimates of the maximum and median activity within the tumours. In order to quantitatively assess the performance of the CNN on tumours split into halves, we measured the ratio between the median activity values measured in each tumour half and compared the results. The CNN underestimated the activity ratio between the two tumour halves in 85% of the test cases. When tumours smaller than 5 ml were excluded from the analysis, the ratio between the median activities measured in the two tumour halves was underestimated by the CNN in 90% of the cases.

**Table 1.** Average RC_max_, RC_median_ and RC_peak_ values relative to experiment 1, where the reconstructed PET images were generated with one PSF. These values are measured only for tumours larger than 5 ml.

Volume ≥ 5 ml	RC_max_	RC_median_	RC_peak_
Reconstructed PET	1.87 ± 0.22	0.86 ± 0.07	1.08 ± 0.05
CNN’s predictions	1.06 ± 0.07	0.91 ± 0.05	0.96 ± 0.04

To describe the smaller volumes we performed an average RC measurement using tumours with volume spanning 1 to 2 ml. To calculate this value the chosen 1 ml interval is split in four smaller segments. The average RC is measured for each segment and subsequently an overall value is obtained by averaging these four RCs. The maximum, median and peak average RCs are measured both using the reconstructed PET images and the CNN predictions, and the results are summarised in table 2.

**Table 2.** Average RC_max_, RC_median_ and RC_peak_ values relative to experiment 1, where the reconstructed PET images were generated with one PSF. These values are measured only for tumour volumes between 1 and 2 ml.

1 ml ≥ volume ≥ 2 ml	RC_max_	RC_median_	RC_peak_
Reconstructed PET	1.46 ± 0.17	0.55 ± 0.06	0.68 ± 0.09
CNN’s predictions	0.81 ± 0.11	0.67 ± 0.13	0.73 ± 0.08

**Figure 6.**
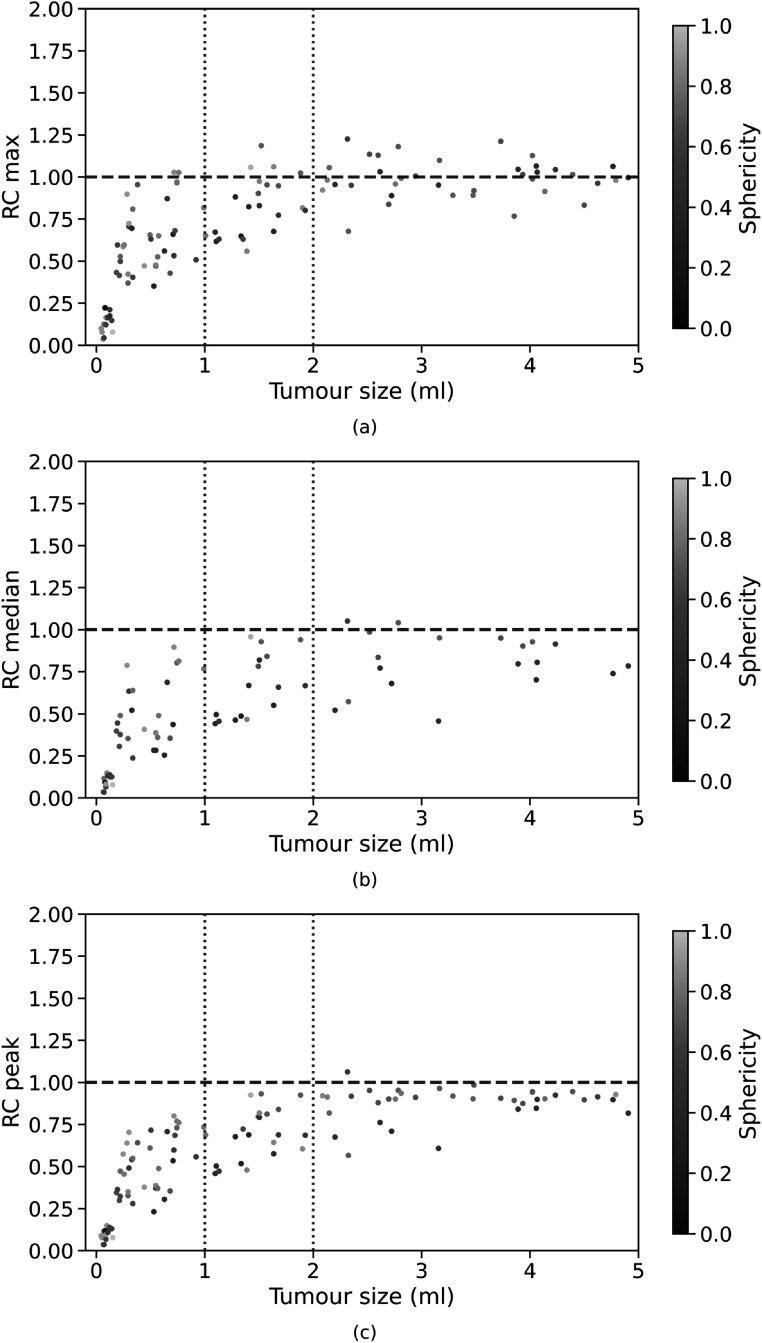
Analysis of tumour volumes smaller than 5 ml predicted by the CNN. The RC_max_, RC_median_ and RC_peak_ values, obtained training and testing the network on PET data generated with a single PSF, are plotted against the tumour volume in (a), (b) and (c) respectively. The tumour sphericity is colour coded using the diverging colour scale shown on the right.

Although the RC values predicted by the CNN for small tumours are lower than 1, the CNN yields improved estimates for all RC. We further investigated which factors affected the recovery of radiotracer activity in the simulated tumours. As the simulated tumours may be characterised by elongated shapes, one parameter that we chose for the analysis of small tumours was the sphericity, a dimensionless metric defined as:

\begin{eqnarray*}{\mathrm{sphericity}}=\displaystyle \frac{\sqrt[3]{36\pi \cdot {V}^{2}}}{A},\end{eqnarray*}

where *V* is the tumour volume and *A* the tumour surface area. The sphericity describes the roundness of the tumour shape relative to a sphere and its values range from 0 to 1. A sphericity value of 1 indicates that the tumour has a perfect spherical shape, a low sphericity value means that the tumour shape is more elongated. In figure 6 the RC values are colour coded based on the sphericity of the corresponding tumour volumes. In these plots, spherical tumours are shown in yellow whereas more elongated tumours are shown in blue. The plots show that higher RCs are often associated with a higher sphericity. This may be due to the fact that elongated tumours are more affected by PVE as a larger fraction of tumour voxels is located closer to the tumour edges and is thus more prone to spilling-in and spilling-out (Soret *et al* 2007). For this reason, it may be more difficult for the CNN to recover the ground truth tumour shape and uptake.

### Application to PET data generated with different PSFs

**Figure 7.**
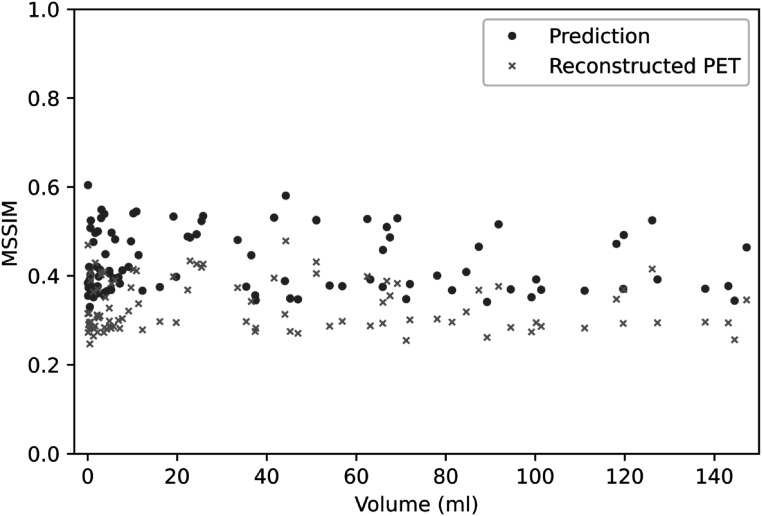
This figure shows all the MSSIM values, measured in the test dataset generated with multiple PSFs. The MSSIMs between reconstructed PET images and ground truth images are shown in orange, the MSSIMs between predictions and ground truth are plotted in blue.

**Figure 8.**
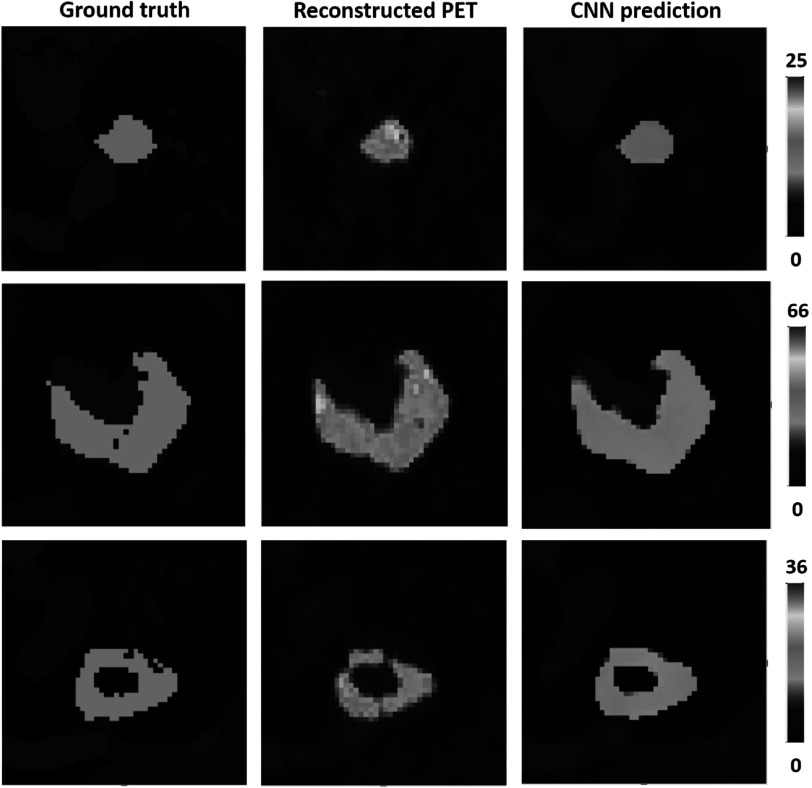
Transverse views of three representative volumes, generated each with a different PSF. The PET images were generated with a PSF with FWHM (4.7, 4.7, 4.2) mm in the first row, a PSF with FWHM (4.0, 4.0, 3.6) mm in the second row and PSF with FWHM (4.5, 4.5, 4.0) mm in the third row. Each column shows the ground truth images, the reconstructed PET images and the CNN’s predicted images respectively. In each row the images are shown with the same colour scale, expressed in kBq ml^−1^.

**Figure 9.**
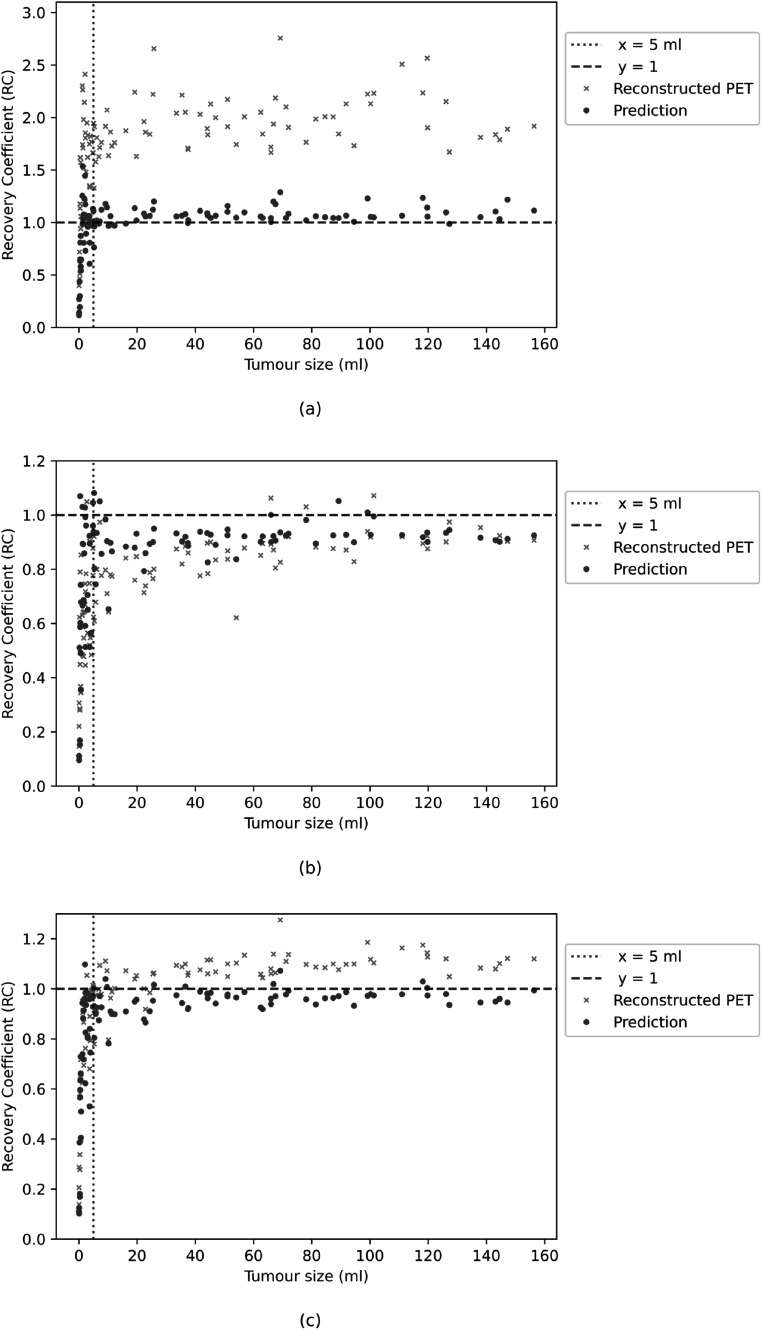
The RC_max_ and RC_median_ values, obtained training and testing the network on PET data generated with a range of PSFs, are plotted against the tumour volume in (a) and (b) respectively. The RC_peak_ values measured on the same dataset are plotted in (c). The coefficients measured using the reconstructed PET images are shown in orange, whereas the ones measured using the predicted images are shown in blue. Tumours split in halves, each assigned with a different activity, were excluded from the calculation of the median values.

**Figure 10.**
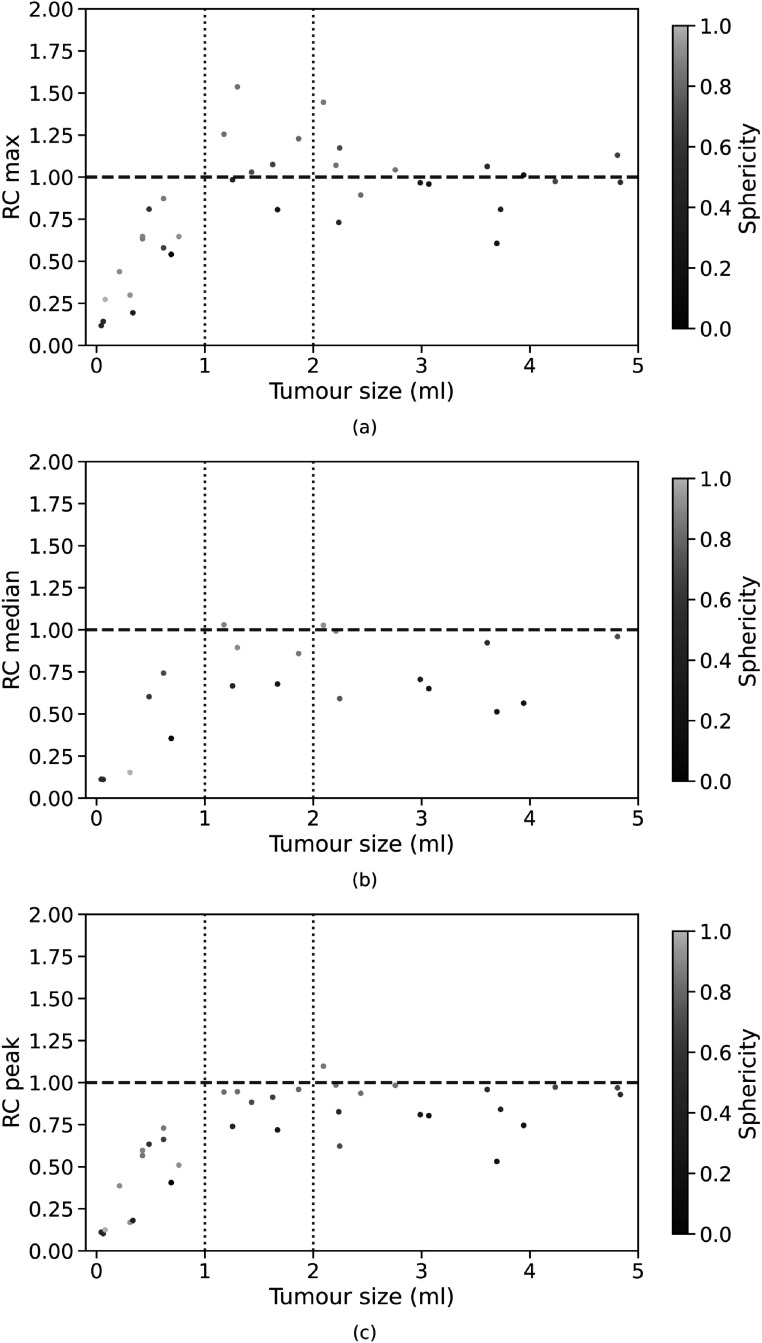
Analysis of tumour volumes smaller than 5 ml predicted by the CNN. The RC_max_, RC_median_ and RC_peak_ values, obtained testing the network on PET data generated with a range of PSFs, are plotted against the tumour volume in (a), (b) and (c) respectively. The tumour sphericity is colour coded using the diverging colour scale shown on the right.

The network previously trained in experiment 1, where the reconstructed PET images were generated with one PSF, was then applied to PET data generated with different PSFs. The MSSIM values measured using the reconstructed PET images and the images predicted by the CNN are shown in figure 7. The average MSSIM measure between the reconstructed PET images and the ground truth images is 0.34 ± 0.07 whereas the average MSSIM between the CNN predictions and the ground truth images is 0.43 ± 0.07. This value is comparable to the MSSIM obtained in the previous experiment. In figure 8, three volumes belonging to the test dataset are presented. The PSFs used to simulate the PET raw data and to reconstruct the PET images had FWHM (4.7, 4.7, 4.2) mm for the images in the first row, FWHM (4.0, 4.0, 3.6) mm for the images in the second row and FWHM (4.5, 4.5, 4.0) mm in the third row. Even though the PSFs used in the first and second row did not match the PSF used to generate and reconstruct the PET images used for training, the network was still able to predict improved tumour shapes and activities. In figure 9 the RC_max_, RC_median_ and RC_peak_ are plotted against the tumour volume expressed in ml. The recovery curves show similar behaviours as in the previous experiment and improved values are obtained when estimating the RCs on the images predicted by the CNNs. The average RC measurements calculated for tumour volumes larger than 5 ml are presented in table 3. The CNN yields improved RCs. The RC estimates shown in this table are comparable to the ones obtained in the previous experiment, showing that the network can successfully recover the maximum, median and peak activity in the tumours even when tested on images generated with different PSF values. A separate analysis was performed for tumour volumes smaller than 5 ml. Average RCs were measured for tumour volumes between 1 and 2 ml as in the previous experiment, and the results are presented in table 4. Although the recovery of small tumours was less accurate, the CNN always yielded improved RC estimates. Again, we measured the sphericity of the small tumours comprised between 1 and 2 ml and figure 10 shows the RC_max_, RC_median_ and RC_peak_ measurements which are colour coded based on the tumour sphericity. Looking at the plots we can observe that in this dataset a lower RC is often associated with lower sphericity, similarly to what we found in the previous experiment.

**Table 3.** Average RC_max_, RC_median_ and RC_peak_ values relative to PET data generated with different PSFs. These values are averaged over tumours with volume larger than 5 ml.

Volume ≥ 5 ml	RC_max_	RC_median_	RC_peak_
Reconstructed PET	1.94 ± 0.26	0.84 ± 0.08	1.07 ± 0.08
CNN’s predictions	1.07 ± 0.08	0.90 ± 0.05	0.95 ± 0.05

**Table 4.** Average RC_max_, RC_median_ and RC_peak_ values obtained testing the network on the reconstructed PET images generated with a range of PSFs. These values are measured only for tumour volumes between 1 and 2 ml.

1 ml ≥ volume ≥ 2 ml	RC_max_	RC_median_	RC_peak_
Reconstructed PET	2.03 ± 0.21	0.59 ± 0.05	0.87 ± 0.07
CNN’s predictions	1.15 ± 0.12	0.84 ± 0.13	0.89 ± 0.06

### Application to PET data generated with different noise levels

As a proof of concept, we performed an experiment using simulated PET images generated with different number of counts. In this case, a small dataset composed of 10 hollow tumours was used as ground truth. The two datasets were generated by setting the number of total prompts to 50 millions and to 200 millions respectively. The CNN that was trained in experiment 1, using PET images generated with 100 million total prompt events, was then applied to the two datasets.

**Figure 11.**
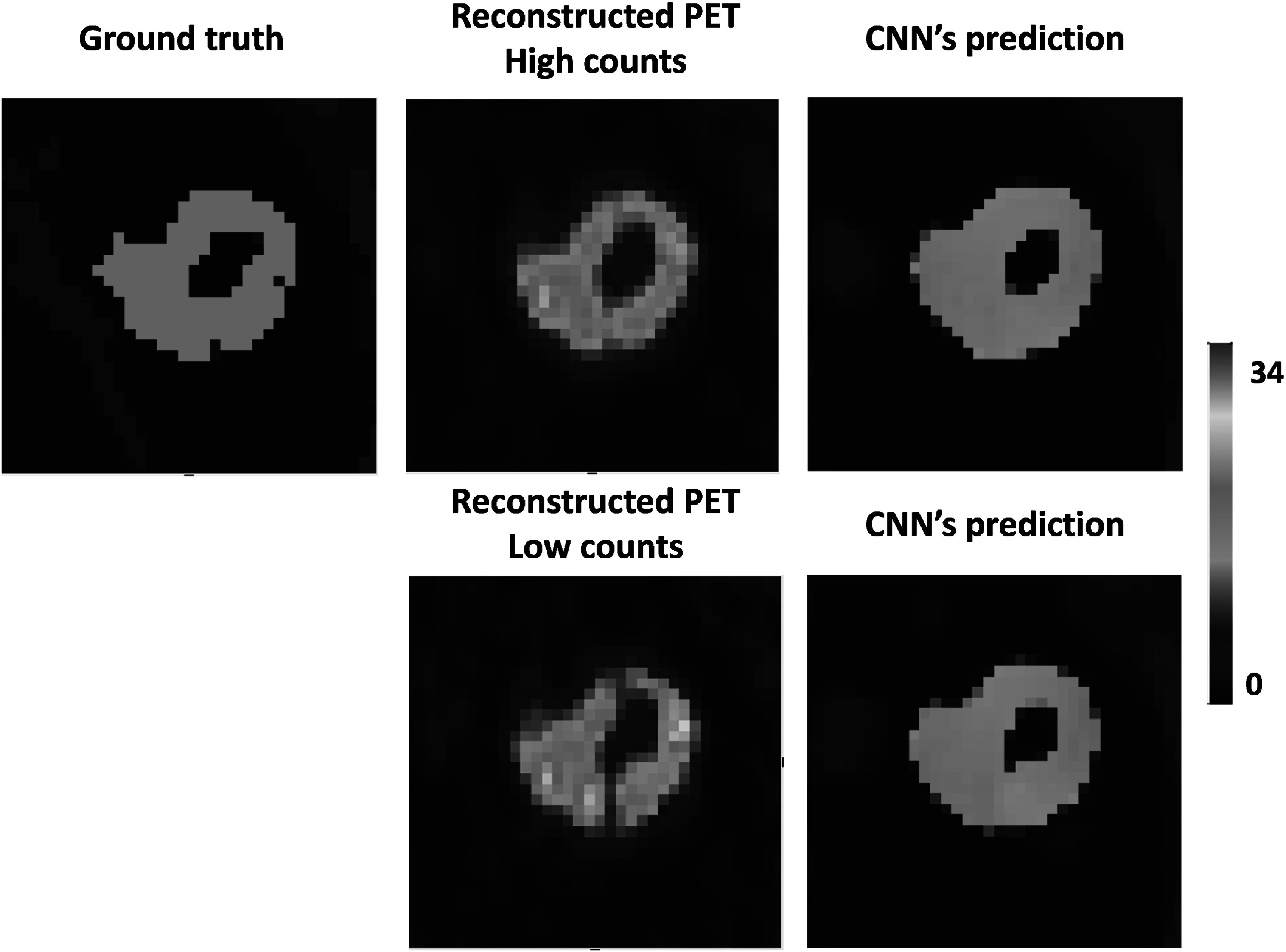
Transverse views of a hollow tumour. The reconstructed PET images in the top row were generated by setting the number of prompt counts to 200 million, the ones in the bottom row were generated using 50 million prompt counts. The CNN predictions are shown in the third column. All the images are shown with the same colour scale, expressed in kBq ml^−1^.

A representative example of a hollow tumour simulated with different number of counts is presented in figure 11. When the CNN was applied to the reconstructed PET images generated with high statistics and with low statistics, it yielded a denoised image with a better defined tumour shape and a more uniform tumour activity distribution.

To quantitatively assess our results, the RCs were measured using the reconstructed PET images and the CNN predictions. As shown in table 5, when applied to the PET images generated with low statistics, the CNN yielded improved RCs. The RCs measured using the reconstructed PET images generated with a high number of counts are presented in table 6. Again, the CNN was able to recover improved RCs. In both cases, the CNN’s performance is comparable to the one obtained in experiment 1, when the CNN was trained and tested on data generated with the same noise level.

**Table 5.** Average RC_max_, RC_median_ and RC_peak_ values obtained testing the network on the reconstructed PET images generated with 50 million counts.

*V* ≥ 5 ml	RC_max_	RC_median_	RC_peak_
Reconstructed PET	2.28 ± 0.32	0.79 ± 0.05	1.08 ± 0.09
CNN’s predictions	1.16 ± 0.07	0.93 ± 0.04	1.00 ± 0.07

**Table 6.** Average RC_max_, RC_median_ and RC_peak_ values obtained testing the network on the reconstructed PET images generated with 200 million counts.

*V* ≥ 5 ml	RC_max_	RC_median_	RC_peak_
Reconstructed PET	1.73 ± 0.14	0.82 ± 0.06	1.04 ± 0.08
CNN’s predictions	1.06 ± 0.05	0.91 ± 0.04	0.96 ± 0.04

## Discussion and conclusion

In this paper we propose a deep learning approach to improve quantification of radiotracer uptake and tumour shape definition in PET images. A 3D CNN was successfully trained and tested on simulated data generated with a single PSF and applied to reconstructed PET images generated with a range of PSFs. The results indicate that the network is able to improve the definition of the tumour shapes and to denoise reconstructed PET images. A quantitative analysis of the results obtained using simulated data has shown that the images predicted by the 3D CNN yield improved estimates of the maximum tumour activities. We observed that the peak, maximum and median activities were not accurately recovered for tumours with volumes smaller than 5 ml, so a more detailed analysis was performed on small tumours. Only tumour volumes smaller than 0.18 ml presented critical issues. Bigger volumes located close to other background structures and characterised by a low ground truth activity were generally associated with an inaccurate prediction of the peak, maximum and median activity. We measured the average RCs for tumours with volumes between 1 and 2 ml and the CNN yielded improved estimates for all RCs. We additionally measured the sphericity for each of these tumours and we observed that lower RCs were often related to a lower tumour sphericity. In future work, we plan to train the network on a bigger dataset and to further augment the training dataset, thus adding more small volumes to the training dataset. This might improve the performance of the network for this class of tumours, as the network would be able to learn from more small tumours during training. A secondary effect that we noticed in our experiments was an improvement in the recovery of background structures. This effect will be further investigated in future work. Our approach proved successful when the network was applied to a set of reconstructed PET images generated with a range of PSFs that did not match the PSF used to generate the training dataset. Preliminary results suggest that the proposed approach would be able to restore PET images acquired with different scanners and spatially varying PSF. Finally, a proof of concept experiment was performed applying the CNN on reconstructed PET images generated with two different noise levels. In both cases, the CNN’s performance is comparable to the one obtained when the CNN was trained and tested on data generate with the same noise levels. This work has the potential to be extended to larger areas of the body, in order to improve the estimation of the total tumour burden. The proposed approach has been tested on images generated with two specific noise levels, the robustness of this method to data generated with other noise levels remains to be evaluated. Before this algorithm can be applied widely, it will be necessary to evaluate its performance on PET images reconstructed with a wider range of number of OSEM iterations and imaging situations in order to determine the range of acquisition, reconstruction and imaging conditions under which it remains valid. In this work the same spatially invariant PSF was used for the simulation of the PET raw data and for the reconstruction of PET images. On the contrary, in a clinical setting PET images would be characterised by spatially variant and non stationary PSFs and there may be a mismatch between the system’s PSF and the one modelled in the reconstruction algorithm. These aspects will be investigated in future work. Further experimentation is also needed to assess the robustness of the proposed deep learning approach to data generated using PSFs characterised by a different anisotropy and to more complex tumour shapes associated with heterogeneous activities. In future work, we plan to extend the proposed approach so that it can be applied to a wider region of the torso.
